# Supplementary material for: Risky decision-making and nonsuicidal self-injury among university students: Examining the role of criticism feedback
Source: PLoS One. 2024 Oct 25;19(10):e0312081. doi: 10.1371/journal.pone.0312081 (PMC11508084; doi:10.1371/journal.pone.0312081)
Supplement: S1 Appendix — (DOCX) [file pone.0312081.s001.docx]

**Appendix A**

The significant block x task x trait self-criticism interaction appeared to be the product of a suppression effect in our model. Results should be interpreted with the caution that this interaction was nonsignificant in a model that did not include the block x task x past-month NSSI interaction. Nevertheless, for the sake of completeness, we followed up the block x task x trait self-criticism interaction using the Johnson-Neyman technique to determine the regions of significance. For the IGT, the simple block slope was significant and negative at SRS scores ≤ 51 (97.55% of the sample). Participants with SRS scores ≥ 52 (2.45% of the sample) did not significantly increase or decrease their risky decision-making across blocks of the IGT. For the CGT, the simple block slope was significant and negative at SRS scores ≥ 24 (75.87% of the sample). Participants with SRS scores ≤ 23 (24.13% of the sample) did not significantly increase or decrease their risky decision-making across blocks of the CGT. Overall, the findings suggest that when controlling for the effect of past-month NSSI, SRS scores were differentially associated with performance on the IGT and CGT across blocks. On both tasks, most people significantly decreased their risky decision-making over time. On the IGT, people with very high trait self-criticism did not improve their performance whereas on the CGT, people with very low trait self-criticism did not improve their performance.
