# Supplementary material for: Risky decision-making and nonsuicidal self-injury among university students: Examining the role of criticism feedback
Source: PLoS One. 2024 Oct 25;19(10):e0312081. doi: 10.1371/journal.pone.0312081 (PMC11508084; doi:10.1371/journal.pone.0312081)
Supplement: Attachment — Submitted filename: NancekivellEtAl_PLOSONE_ResponseLetter.docx [file pone.0312081.s002.docx]

Gaëtan Merlhiot, PhD

Academic Editor, *PLOS ONE*

mobiLAB (VEDECOM)
23 bis allée des Marronniers
78000 Versailles

Dear Dr. Merlhiot,

Thank you for your evaluation and to the reviewers for their constructive comments. The Reviewers’ feedback was encouraging, and all identified some of the manuscript’s strengths. Specifically, ***Reviewer 1*** one stated that the paper is “*technically well done and the report is clearly written*” an also noted that the work “*adds importantly to the literature on NSSI and risky decision making and points to potentially useful directions for further work on the topic*”. ***Reviewer 2*** highlighted similar strengths to Reviewer 1: “*this is a well-written manuscript describing the results of a well-designed experiment investigating a topic of high clinical relevance.*” Finally, ***Reviewer 3*** appreciated that the study “*is well-designed*” and that we “*followed the pre-defined research plan*.”

We have responded to the Reviewers’ comments below. We believe the review process has further strengthened our submission. We thank you for your time and efforts in considering this revised manuscript for publication in *PLOS ONE*.

**Editor**

1. **Please ensure that your manuscript meets PLOS ONE's style requirements, including those for file naming. The PLOS ONE style templates can be found at** https://journals.plos.org/plosone/s/file?id=wjVg/PLOSOne_formatting_sample_main_body.pdf **and** https://journals.plos.org/plosone/s/file?id=ba62/PLOSOne_formatting_sample_title_authors_affiliations.pdf

We have reviewed the style requirements, and the revised submission is consistent with these requirements.

2. **Thank you for uploading your study's underlying data set. Unfortunately, the repository you have noted in your Data Availability statement does not qualify as an acceptable data repository according to PLOS's standards.**

**At this time, please upload the minimal data set necessary to replicate your study's findings to a stable, public repository (such as figshare or Dryad) and provide us with the relevant URLs, DOIs, or accession numbers that may be used to access these data. For a list of recommended repositories and additional information on PLOS standards for data deposition, please see** <https://journals.plos.org/plosone/s/recommended-repositories>**.**

We have uploaded the minimal dataset necessary to replicate our study’s findings to the existing Open Science Framework page: <https://osf.io/fctq5/>. We have updated the URL in the manuscript to direct readers to the updated page. This meets PLOS’s standards for making the data available.

3. **Please include your full ethics statement in the ‘Methods’ section of your manuscript file. In your statement, please include the full name of the IRB or ethics committee who approved or waived your study, as well as whether or not you obtained informed written or verbal consent. If consent was waived for your study, please include this information in your statement as well.**

We have added the full ethics statement in the ‘Methods’ section, including the full name of the ethics committee who waived our study, and the form of consent obtained (written).

**Reviewer 1**

1. **As the authors themselves identify, their manipulation of the criticism variable introduced a confounding of message content with the occurrence of a message. It may be that receiving a message while completing the gambling task distracts the participant irrespective of its content and this leads to poorer performance by those with higher frequencies of NSSI. This interpretation requires of course that higher frequency NSSI is linked to a greater propensity for distraction. This strikes me as a plausible rival hypothesis to sensitivity to criticism and as such should be at least entertained in the Discussion.**

We thank Reviewer 1 for raising this important point. We agree that the consequences of the methodological limitations of the study needed to be more deeply examined in the ‘Discussion’. Having 20 seconds of audio preceding blocks in the CGT but not the IGT introduces plausible alternative explanations for our findings, and we now touch on two of them in the ‘Discussion’ (‘Limitations’, p. 27):

*“Fourth, in the CGT, participants heard 20-second audio clips at the beginning of each block (6 total), but the IGT did not have any audio preceding the blocks. Accordingly, the tasks differed in the presence versus absence of audio messages in general. Our effects may have occurred because criticism, specifically, impacts decision-making among people with recent NSSI, but our design cannot rule out some alternative explanations. For instance, relative to the CGT, the IGT had a lower minimum time between blocks. Greater working memory capacity is associated with better performance on the IGT because working memory predicts being certain of which decks are “good” and which are “bad” (i.e., performance in the conceptual period) (126, 127; although see 128 for contradictory findings). Having a 20-second pause may have introduced a greater working memory load on the CGT relative to the IGT. Relatedly, the CGT may have been more distracting than the IGT; thus, greater proneness to distraction among people with recent NSSI (e.g., 129) could have partially explained the link between NSSI and riskier decision-making on the CGT. However, trait impulsivity is associated with both lower performance on working memory tasks (e.g., 130) and proneness to distraction (e.g., 131,132). We found that the interactions involving past-month NSSI in our models persisted when the effects of trait impulsivity were included. Nonetheless, future studies should use versions of the IGT that have non-critical audio feedback (e.g., neutral or positive) matched for duration to the CGT to support stronger inferences about the unique role criticism might play in how NSSI impacts risky decision-making.”*

2. **Crucial to the conclusion reached on the basis of the analysis is that the slopes of the regression lines for the standard gambling task and the gambling task with added criticism differ (p.15, ll. 15-17). How do we know that one is “significantly stronger” that the other? Is it based on a judgement by eye? Is it based on the fact that one is statistically significant and the other is not? Or is there a test of significance of the difference that is not reported. Without the latter, some justification for the inference needs to be provided.**

The test of significance of the difference is the interaction effect itself (*b*, *t*, and *p* reported in the sentence preceding simple slopes results). That is, the *t* test for the difference between the slopes is exactly the same as the *t* test for the regression coefficient for the two-way interaction term in the overall analysis. Thus, if the two-way interaction is significant, then all the simple slope lines are significantly different from each other, and “no further test is required of whether the simple slopes of Y on X differ from one another as a function of Z [moderator]” (Aiken & West (1991, pp. 19-21; see also Hayes, 2018, pp. 261-262).

3. **A total of 23% of the original sample did not provide data for the analysis. I expected some comment on this, but may have missed it.**

Studies have shown that online administrations of the IGT (and other tasks capturing impulsive behaviors) are feasible and show good convergent validity with tasks designed to assess processes underlying IGT performance administered in lab (Verdejo-Garcia et al., 2021, *Nature Human Behavior*). Because of the online nature of the study, however, we had a large percentage of missingness, which reduced our sample size. We believe there are two main sources of attrition. First, the study involved switching between two separate online platforms (Qualtrics for questionnaires and Inquisit for IGT/CGT). Participants who completed the questionnaires were also sent a link to Inquisit. Despite consenting, 17 participants did not access the portion of the study where the IGT/CGT were completed. Second, because of the longer task duration (~15 minutes for the IGT/CGT), and participant ability to end participation at their own discretion, 49 participants completed some but not all the tasks. Ultimately, we chose to remove participants with missing IGT/CGT data and/or self-injury history because these variables were needed for primary analyses. Additionally, in most cases participants were missing a considerable amount of data: Of the 66 participants missing any IGT/CGT data, 59 (89.39%) were missing 25% or more data and 46 (69.70%) were missing 50% or more data. Further, the minimum amount of missingness was 14 trials, followed by 19 trials, etc. suggesting that except for 2 participants, all others missing data had at least one full block (i.e., 20 trials) or more of missing data, thereby warranting exclusion from primary analyses.

We have added information about missing data and participants who were removed from analyses in the ‘Participants’ section (pp. 8-9). We have also raised missing data as a limitation in our ‘Discussion’ section (p. 26-27).

4. **Why was frequency of NSSI in the past month selected as the NSSI measure? Would use of any of the other NSSI measures produced the same result?**

We selected past-month NSSI because we were interested in the frequency of recent NSSI. Past-week NSSI, although a more recent indicator than past-month, had relatively low counts both in terms of participants (29/286 endorsing past-week NSSI) and episodes (0-7 episodes, with only 10 people endorsing >1 NSSI episode in the past week). Conversely, past-year NSSI was not adequately “recent” for our research questions. Past-month NSSI was methodologically and conceptually appropriate. Indeed, research has supported past-month NSSI, specifically, as an important predictor of psychological (e.g., post-hospitalization persistence of NSSI, Mason et al., 2024, *Journal of Child Psychology and Psychiatry*; Kaplan et al., 2016, *Comprehensive Psychiatry*; rehospitalization, van Alphen et al., 2017, *Journal of Clinical Psychiatry*; symptom severity (e.g., depression, borderline personality disorder;, Christensen et al., 2021, *Journal of Psychiatric Research*) and psychosocial (e.g., borderline personality disorder symptom severity, Kaplan et al., 2016; less positive health behaviours, Jarvi et al., 2017, *Journal of Clinical Psychology*) outcomes. We suspect that past-week NSSI may also produce the same result, although it is possible that due low counts the result would be nonsignificant and/or spurious. Past-year and lifetime NSSI could also produce the same result, unless the effects observed occur only in the context of recent NSSI (i.e., risky decision-making waxes and wanes over periods of a year or longer).

We have added the rationale to the ‘Introduction’ (p. 7) and the ‘Measures’ section (p.13). We did not test all iterations of the NSSI outcome variable in our model because we did not want to unnecessarily inflate family-wise error, and furthermore, an analysis of all NSSI time frames would have produced a cumbersome manuscript. We provide access to the data used in analyses so that scholars may test other NSSI timeframes if they have hypotheses about these relations that differ from ours (see <https://osf.io/fctq5/>).

5. **The skewness estimates for the variables listed in Table 2 differ substantially. Would this have had any effect on the correlations summarised in the table?**

Generally, reputable sources note that, at least for descriptive use, differential skewness in variables is not a concern for correlations. For example, in their landmark text, Cohen, Cohen, Aiken, & West (2003) write “no assumptions are necessary for the computation of correlation, regression, and other associated coefficients” (p. 41). When using statistics inferentially (our use in the paper is descriptive), the authors continue to say that, although there are certain assumptions in these cases, “fortunately, these statistics are robust; that is, moderate departure from these assumptions will usually result in little error of inference.” (p. 41).

Nonetheless, we were wary of the fact that Pearson’s *r* correlations can be attenuated in cases where two variables have substantially different distributions. To make sure that this did not happen, we chose a non-parametric correlation (Spearman *ρ*) because it uses ranks instead of distributional information. Therefore, the correlations in the table were not attenuated by variables with different distributions (i.e., different skewness values, as Reviewer 1 pointed out).

6. **Is the final N for analysis 286 or 284. My addition of numbers in Table 1 indicates the latter.**

The final N is 286. As indicated in the note for Table 1, two participants were missing gender data, which is why the N based on ‘Gender’ is 284 rather than 286. Similarly, N based on ‘Race/Ethnicity’ is 281 due to three participants preferring to not indicate their race/ethnicity and two participants missing race/ethnicity data.

7. **A correlation of .12 in Table 2 is both statistically significant and non-significant depending on the variables correlated. Does this mean that the Ns differ across variables or that the asterisk has been accidentally omitted for one of the correlations?**

We thank Reviewer 1 for the opportunity to clarify this. The correlations presented in Table 2 are rounded to two decimal places, which is consistent with how we handle statistics throughout the manuscript. Therefore, although they seem to be the exact same values, they are not.

The exact value for the correlation between “Past-year NSSI” and “CGT average” is 0.119822 and that correlation is statistically significant, *p*<0.05 (the *N* is 277 for that analysis as well, which impacts *p*-values).

The exact value for the correlation between “Lifetime SA” and “Past-month NSSI” is 0.12006 which is statistically significant (*p*<0.05; exact *p*=0.0425). In contrast, the exact value for the correlation between “Lifetime SA” and “Past-week NSSI” is 0.115075, and this is non-significant (exact *p*=0.0519).

In sum, 3 of the correlation values presented in Table 2 were correctly rounded to 0.12. However, they had slightly different exact values, and the lowest exact value did not value below the alpha cutoff (p<0.05) we were using to denote statistical significance.

8. **Table 2 provides only one statistic for the SA variable, apart from the correlations. Is there a reason for this? And it is not clear what the statistic that is provided is an estimate of. Is it the average number of suicide attempts across the 286 (284) participants, which seems very high, or is it the total number of attempts for the sample? A median and interquartile range might be a better way of summarising the data for this variable.**

We have added a note below Tables 1 and 2 to clarify that 67 refers to the number of participants who reported at least one lifetime suicide attempt. The SITBI-R (Fox et al., 2020, *Psychological Assessment*) does not explicitly collect information about the number of lifetime SA. Further, we were interested in lifetime SA only as a rival clinical predictor to NSSI, and thus the binary presence/absence was appropriate for our purposes. Supporting this decision, previous work has shown that in undergraduate samples, 1 is the most common number of attempts among university students with lifetime SA history (Sivertsen et al., 2019, *BJPsych Open*).

**Reviewer 2**

1. **P. 3, lines 10-11: This statement seemed a bit of an oversimplification of understandings of NSSI. For example, the Benefits/Barriers Model (Franklin & Hooley, 2018) could be worth mentioning to provide a bit more nuance.**

We have added more context before the statement (p. 3). We now note that NSSI is an emotionally and physically costly behavior and involves overcoming a fundamental drive to avoid pain. It was thus historically viewed that NSSI involves emotional reactivity and an inability to resist urges to engage in NSSI.

2. **P. 8, line 4: Please provide more information on the online advertisements to help better characterize the sample. Where were these advertisements posted? Were there any other inclusion/exclusion criteria applied to those recruited online vs the course credit pool?**

We have provided more information on the online advertisements (i.e., where they were posted), and clarified that inclusion/exclusion criteria did not vary based on recruitment avenue (i.e., people recruited online versus the course credit pool). These details are in the ‘Participants’ section (p. 8).

3. **P. 13, line 20: It would be useful to clarify again that NSSI values referred to behavior frequency over those timeframes (versus dichotomous variables).**

We have clarified that NSSI values refer to behavior frequency. See description on pp. 12-13 and p. 15.

4. **P. 21, line 23: Please be sure to use person-first language.**

We have updated language to be first-person: “people with versus without prior attempts” instead of “attempters and non-attempters." (p. 25). This sentence was on p. 21 (line 23) of the initial submission as Reviewer 2 noted.

5. **Was there any effort to ascertain how salient participants found the critical statements/how much they identified with the topics? Another explanation for the relatively small effects is that certain participants found the statements harder to identify with and thus experienced them as less threatening.**

Thank you for raising this important issue. We did not directly measure how much participants identified with the topics that were raised in the audio criticism on the CGT. We agree that potential individual differences in the relevance of the criticism leveled against participants could have impacted the magnitude of our estimates of the effect of NSSI behavior on risky decisions (plays on disadvantageous decks) on the CGT. Curiously, however, a study that examined adolescents’ ratings of the applicability of negative parental feedback found that that negative feedback that was *less* personally applicable was associated with *greater increases* in negative affect (van Houtum et al., 2022). Further, although not directly related to the reviewer’s emphasis on participants’ identification with statements, some studies suggest that adolescents’ perceptions of parental criticism (i.e., how critical their parent is of them) are not associated with their emotional and neural responses to parental criticism in experimental contexts (e.g., Bonduelle et al., 2023, *Journal of Experimental Child Psychology*; van Houtum et al., 2022, Developmental Cognitive Neuroscience). Overall, the empirical findings on how much personal relevance moderates the impact of criticism on mood and cognition are likely mixed.

With that said, we made several changes to the manuscript to address Reviewer 2’s important observation. First, in our description of the CGT, we have added information to support the validity of the audio criticism in evoking a robust emotional response – potentially indicative of feeling criticized – for adult participants on average. We note that the audio stimuli were developed based on actual brief recordings of parents giving critical feedback to their daughters (Hooley et al., 2005, *Biological Psychiatry*; Hooley et al., 2009, *Psychiatry Research: Neuroimaging*). We used audio previously used by Allen and colleagues (2019) that produced significant changes in negative affect in two studies. Critically, changes in negative affect were invariant across NSSI groups. We highlight this information on p. 12.

Second, we note in our discussion that, although we did not directly measure how much participants identified with the critical statements, we measured trait self-criticism using the Self-Criticism Rating Scale (SRS) in our rival predictor analyses. As we describe in the manuscript (p. 22), a proposed mechanism through which audio criticism affects decision-making is by activating trait self-criticism. Through this logic, individual differences in trait self-criticism (SRS scores) would have a substantial influence on how the audio criticism impacted performance. Further, perceptions of criticism from others (e.g., parents) and trait self-criticism are correlated (Masland et al., 2019, *Journal of Family Psychology*); indeed, trait self-criticism is hypothesized to develop partially through the internalization of criticism and other negative interactions with key attachment figures, particularly parents (Beck, 2008, *American Journal of Psychiatry*; Naismith et al., 2018, *Clinical Psychology and Psychotherapy*). Taken together, although we did not directly measure how much participants identified with the criticism or how personal it was, we contend that SRS analyses may address this indirectly. When NSSI was included in the model, SRS scores were associated with patterns of risky decision-making on our tasks, although we interpret this as a suppression effect (see S1 Appendix and pp. 18-19). Regardless, the 3-way interaction involving NSSI persisted when trait self-criticism was in the model; this is indirect evidence that our NSSI results were not significantly impacted by how threatening participants viewed the audio criticism. We have added these details on p. 25.

Third, we acknowledge that not directly knowing how much participants identified with the criticism is a limitation of our methodological approach (p. 28). We note that a promising future direction is to adopt an approach like Hooley and colleagues (2005, 2009) neuroimaging work wherein audio criticism was recorded by the participants’ loved ones and were individualized to the participants’ characteristics and behaviors.

6. **If space allows, it would be helpful to depict the interaction results in a table to make them easier to read.**

We thank Review 2 for this feedback, which we carefully considered. Per APA style, tables can/should be used to present a large amount of information in a comprehensible way (<https://apastyle.apa.org/style-grammar-guidelines/tables-figures>), generally when information presented in a table is clearer than it would be in the main text. We did not feel that presenting interaction results in tables would yield greater comprehension or clarity. For primary analyses (task x block interaction; task x block x past-month NSSI interaction) we were only interested in the interaction effect itself (we did not interpret lower-order effects). Thus, we felt it would be redundant to only present one line of results (interaction effect) in a table, and conversely, unnecessary to present lower-order effects when we did not interpret them.

In terms of presenting the follow-up results of interactions: Aside from the task x block interaction, which was followed up with simple slopes analyses, we otherwise used Johnson-Neyman (which produces results not easily or productively amenable to tables). Indeed, Johnson-Neyman produces ranges of significance which we feel were better explained in-text with supporting figures.

Finally, for analyses testing rival clinical predictors (which we view as ancillary to primary analyses), we were most interested in statistical significance. Specifically, whether the effect of past-month NSSI persisted above and beyond rival clinical predictors in our model, and whether any rival clinical predictors themselves were significant. We felt dissemination of these results was best achieved through concisely presented p-values (both individual and ranges).

Therefore, while we agree that in many cases, interaction results are best presented in a table, in the context of our study and given relevant considerations above, we feel that presentation in-text is the appropriate choice that allows the most comprehensible, clear, and concise dissemination of our results.

7. **Fig 2 - please revise per APA style to make easier to read.**

We agree that Figure 2 could be more reader-friendly and made changes that help. Specifically, we revised the x-axis label (previously “Past_Month_NSSI”) to remove underscores and add units of measurement (now “Frequency of past-month NSSI”)

**Reviewer 3**

1. **As it was highlighted in the limitation section, a very specific population was included (i.e. university students). I suggest to highlight this information even in the title of the article.**

We acknowledge the importance of highlighting the specific population used in this study. Accordingly, we have now amended our title (“Risky decision-making and nonsuicidal self-injury among university students: Examining the role of criticism feedback”).

2. **Please provide more information about the recruitment process: e.g. A) how has it been implemented to involve people with higher risk for NSSI, B) what was the target population of online advertisements (ensuring that real persons meeting inclusion criteria have joined to the study).**

We provided more information about the recruitment process, and how it was implemented to involve people at higher risk for NSSI. Note: We recruited participants who had current/past NSSI and/or a past suicide attempt, rather than people at higher risk for self-injury (but possibly without NSSI/SA). The target population of online advertisements was university students at our institution. These details are now provided in the ‘Participants’ section on p. 8.

3. **I also suggest to reconsider the concept of “Table 1”. As it is meant to demonstrate demographical characteristics, participants with no self-injurious behaviour should be highlighted as well. Furthermore, it is not clear how self-injurious behaviours were organized / grouped, since group-numbers are not matched.**

We appreciate Reviewer 3’s feedback and worked to make Table 1 clearer and easier to interpret. We made the title more descriptive, so it would be clear to readers the table provided demographical and self-injury history characteristics of our sample. We also added notes below the table to clarify values for self-injurious behaviors. Specifically, for self-injurious behaviors, we indicate that the timeframe variables (past-week, -month, -year, and lifetime) all refer to the number (*n*) of people who reported NSSI and/or suicide attempt over the specified timeframe.

Since we do not test differences between people with versus without NSSI, a stratified by lifetime NSSI history was inappropriate. Instead, we present information about people endorsing NSSI over each specified timeframe (past-week, -month, -year, and lifetime), which also conveys information about people who *did not* endorse NSSI over these same timeframes.

Please see Table 1 in the revised manuscript for all these changes.

4. **As it is mentioned in the “limitations” section, the IGT version used in this study has no audio feedback (Page 23, Line 10), unlike the CGT version. One of my major concerns is that most studies use the IGT with immediate audio and visual feedback (e.g. happy or sad faces and happy or sad sound effects). These effects can be essential - I recommend to see the literature of the “somatic marker hypothesis”. Using an IGT version with no audio effects could be one of the main methodological issues, limiting the comparability of these results with previous findings in the literature. It may also limit the comparability of the IGT and CGT.**

We appreciate the chance to clarify the statement that was on page 23, line 10 in the original submission. We acknowledge that the phrasing in our limitations section regarding the presence/absence of “audio feedback” was misleading. The use of “feedback” makes it sound like we were referring to the information participants receive after selecting play versus pass on each trial. This is not what we meant to refer to.

We have amended that sentence to read as follows:

*“Fourth, in the CGT, participants heard 20-second audio clips at the beginning of each block (6 total), but the IGT did not have any audio preceding the blocks. Accordingly, the tasks differed in the presence versus absence of audio messages in general.”* (p. 27)

We agree with Reviewer 3 that having the *trial-level feedback* (i.e., input participants get after selecting play or pass) differ between the IGT and CGT would limit compatibility of the results with previous findings from the literature and limit the extent to which we could compare performance on the IGT and CGT in this study. However, in this study, the IGT and CGT were identical aside from the 20-s audio clips that were added to the beginning of the blocks in the CGT. Critically, the tasks had the same type of feedback: visual input on whether participants lost, won, or neither won nor lost money (see “Iowa Gambling Task (IGT) and Criticism Gambling Task” section of “Measures” - p. 11).

We have expanded our comment on having audio clips preceding the blocks in the CGT but not the IGT. We briefly discussed alternative explanations for our effects (e.g., the role of distraction; see response to Reviewer 1, Comment 1) and proposed important future directions (see “Limitations”, pp. 27-28).

5. **It is unclear whether the study measured the impact of "criticism from important others" and not the effect of criticism per se. Information about the voice during the CGT was not provided. If the participant chose their father as the reference person, was the voice matched to this choice, or everyone heard the text in the same way?**

All participants heard a female voice, regardless of the reference person they selected. However, as noted in the Methods (p. 11), nearly all participants (91.61%, n=262) selected their mother as the person they imagined saying the critical comments to them. Nine additional participants (3.15%) selected another female with whom they have a relationship with (e.g., adoptive mother, stepmother, female non-relative). Only four participants (1.40%) selected their father, and 11 participants did not provide an answer. Thus, for nearly all participants, the voice of critical audio and the reference person were congruent.

The audio stimuli – including its content and vocal characteristics - has been used in several studies with increases in negative mood (Allen et al., 2019, *Psychiatry Research*; Dedoncker et al., 2019, *Brain Imaging and Behavior*). The empirical foundations of this methodology were studies involving children and their mothers wherein mothers recorded personal critical comments that were played to their daughters during fMRI sessions (Hooley et al., 2005, *Biological Psychiatry*; Hooley et al., 2009, *Psychiatry Research: Neuroimaging*). We believe that some of this context supports an inference that “criticism from others” may be driving at least part of the overall effect. However, we did not directly measure how much participants identified personally with the critical comments (see our response to Reviewer 2, Comment 5), so it is possible that the effects we observed are driven by an alternate process, like general changes in mood produced by hearing negative information. To address these issues, we have added substantially more nuance to the ‘Discussion’ (p. 21; p. 25) and more fully acknowledge alternative explanations for the effects we observed (see ‘Limitations’, pp. 27-28).

6. **Although, the mental operation “to imagine” (that the source *Evokes changes in affect, evokes feeling criticized* of the critical comment is a loved one person) may not be a sufficient condition to conclude that indeed the effect of the ‘critical comments from important others’ was measured. Please provide more methodological details if this concept can be supported; otherwise, I suggest to use a more cautious formulation in that question.**

We appreciate Reviewer 3 pointing this out and agree that it is a key methodological consideration. Please also see our response to Review 2, Comment 5.

First, in our description of the CGT, we have added information to support the validity of the audio criticism in evoking a robust emotional response – potentially indicative of feeling criticized – for participants on average. We note that the audio stimuli were developed based on actual brief recordings of parents giving critical feedback to their daughters (Hooley et al., 2005, *Biological Psychiatry*; Hooley et al., 2009, *Psychiatry Research: Neuroimaging*). We used stimuli previously used by Allen and colleagues (2019) that produced significant changes in negative affect in two studies. Critically, changes in negative affect were invariant across NSSI groups. We highlight this information on p. 12.

However, we agree with Reviewer 3 that, because we did not directly capture information that would allow us to definitively conclude that our effects were driven specifically by the impact that criticism from close others has on a person. Per Reviewer 3’s suggestions, we provided a more cautious interpretation of the results, that more thoroughly acknowledges limitations of our tasks, and advances alternative explanations that should be tested and ruled out in future research to support our inferences.

We added more nuance to the ‘Criticism and Decision-Making’ section of the ‘Discussion’ (p. 21) wherein we raise the issue of not knowing how much the task represented what it is like for participants to get *in situ* criticism from loved ones. We also acknowledged this as an important limitation of the experimental procedures in this study, and discussed how future studies could be set up to test the impact of criticism from loved ones on risky decision-making more directly (p. 28).

7. **IGT has more versions (e.g. ABCD and EFGH), enabling to study whether participants are sensitive to reward, punishment or have “myopia for future”. Please take into consideration to discuss decision-making functioning in the light of these research findings.**

We appreciate Review 3 raising this point; we believe that it is important to acknowledge how our findings may be relatively specific to how we operationalized risky decision-making. Specifically, we used a payoff structure and format for the IGT/CGT that were in line with the version of the play/pass IGT used by Cauffmann and colleagues (2010). As Reviewer 3 notes, other versions of the IGT are designed to capture distinct processes (e.g., reward and punishment sensitivity) that tend to influence risk decision-making.

Ultimately, our results may not generalize to other version of the IGT that use different payoff structures, such as the EFGH version (designed to be influenced by individual differences in punishment sensitivity). For example, in a study of participants with depression who had made a suicide attempt in the past 72 hours, participants with a recent suicide attempt performed significantly worse on the ABCD version of the IGT compared to healthy controls, but there were no differences on the EFGH version (Hegedűs et al., 2018, *Journal of Affective Disorders*).

We have noted the limits to generalizability of our findings and have discussed the implications of our choice of IGT versions in general in our ‘Discussion’ section (p. 28).

8. **Direct comparison of persons with history of NSSI and persons with no history of NSSI could strengthen that findings regarding the associations of decision-making performance and criticism may be more specific in case of individuals with history of NSSI.**

As we note in the ‘Introduction’, we were particularly interested in the frequency of recent NSSI (p. 7). Indeed, one of the ways in which we advance methodology from past studies (which have looked only at presence or absence of lifetime NSSI) is by considering the recency and frequency of NSSI. Our decision to focus on the frequency of recent NSSI was based on research supporting past-month NSSI, specifically, as an important predictor of psychological (e.g., post-hospitalization persistence of NSSI, Mason et al., 2024, *Journal of Child Psychology and Psychiatry*; Kaplan et al., 2016, *Comprehensive Psychiatry*; rehospitalization, van Alphen et al., 2017, *Journal of Clinical Psychiatry*; symptom severity (e.g., depression, borderline personality disorder), Christensen et al., 2021, *Journal of Psychiatric Research*) and psychosocial (e.g., borderline personality disorder symptom severity, Kaplan et al., 2016; less positive health behaviours, Jarvi et al., 2017, *Journal of Clinical Psychology*) outcomes (see p. 7). We also had practical reasons for focusing on recent NSSI frequency versus more distal NSSI behavior (p. 13; see also our response to Reviewer 1, Comment 4). We did not anticipate that longer-timeframe NSSI variables (such as lifetime NSSI) would have the same association with decision-making in the context of criticism. Supporting this expectation, we ran our model with lifetime NSSI as a binary variable (yes/no); the three-way task x block x lifetime NSSI interaction is nonsignificant.

9. **Some minor grammatical mistakes can be found (punctuation mistakes; unclear sentences, e.g. Page 5, Line 13-14; not precisely used academical English phrases, e.g. “in sum”). Although the text is basically well-written, perhaps a final proofreading would improve the paper's quality.**

We thank Reviewer 3 for this reminder. We have corrected the example on p. 5 that Reviewer 3 referred to. We have also done a careful proofread of the manuscript for grammar, punctuation and awkward sentences.

In addition to the changes we made based on reviewer comments, a few additional changes were made to correct for a clerical data processing error. Specifically, in our original submission, total Self-Criticism Scale scores did not account for all eight items of the measure. We have since corrected the scores, which did not notably impact our originally reported results. Trait self-criticism was tested as a rival clinical predictor against past-month NSSI. In our original submission, we reported that the task x block x past-month NSSI interaction remained significant above and beyond the task x block x SRS interaction in the same model, and the task x block x SRS interaction was nonsignificant. With corrected scores, the effect of past-month NSSI still remains significant with the task x block x SRS interaction. The task x block x SRS interaction, however, is now significant, which appears to be the product of a suppression effect in our model. The task x block x SRS interaction is nonsignificant on its own (i.e., without past-month NSSI interaction), *p* = 0.097. We have updated the results and discussion in accordance with this finding. We do not present or interpret follow-up tests in the main text (see p. 19); for completeness, these are available in S1 Appendix (which we added on this revision).

***

Thank you, again, for the helpful feedback on our manuscript. We believe that addressing the comments above has made the product stronger. We look forward to moving this work towards publication in *PLOS ONE*.

Sincerely,

Jeremy G. Stewart, PhD, C.Psych

Associate Professor

Departments of Psychology, Psychiatry, and Centre for Neuroscience Studies

Queen’s University
